# Gastric cancer with distinct Epstein–Barr virus-positive and -negative tumor components and their whole exome sequencing result: a case Report

**DOI:** 10.1186/s13000-023-01363-3

**Published:** 2023-07-11

**Authors:** Ki Bum Park, An Na Seo, Moonsik Kim

**Affiliations:** 1 Department of Surgery, School of Medicine, Kyungpook National University, Kyungpook National University Chilgok Hospital, Daegu, Republic of Korea; 2 Department of Pathology, School of Medicine, Kyungpook National University, Kyungpook National University Chilgok Hospital, 807 Hogukno, Buk-gu, Daegu, 41404 Republic of Korea

**Keywords:** Epstein–Barr virus, Heterogeneity, Collision tumor, Gastric carcinoma with lymphoid stroma, Whole exome sequencing

## Abstract

**Background:**

Epstein–Barr virus (EBV)-associated gastric cancer exhibits distinct clinicopathologic characteristics, showing a good response to immune checkpoint inhibitors and a favorable prognosis. However, gastric cancer comprising distinct EBV-positive and -negative components in a single mass have been rarely reported, and their detailed genetic characteristics have not yet been investigated. Therefore, we reported the case of gastric cancer exhibiting distinct EBV-positive and -negative areas and further investigated its genetic characteristics.

**Case presentations:**

A 70-year-old man underwent distal gastrectomy for gastric cancer, which was detected during a routine health check-up. EBV-encoded RNA in situ hybridization revealed distinct EBV-positive and -negative components at each other’s borders, morphologically consistent with collision tumor. We separately sequenced EBV-positive and -negative tumor areas through whole exome sequencing (WES) with matched normal tissue. Remarkably, both EBV-positive and -negative areas shared pathogenic mutations of *ARID1A*, *KCNJ2*, and *RRAS2*. Furthermore, they shared 92 somatic single nucleotide variants and small insertion or deletion mutations, of which 32.7% and 24.5% are EBV-positive and -negative tumor components, respectively.

**Conclusions:**

WES results suggested that gastric cancer with distinct EBV-positive and -negative tumor components, formerly categorized as a collision tumor, can be clonally related. EBV-negative tumor component might be associated with loss of EBV during tumor progression.

## Background

Epstein–Barr virus (EBV)-associated gastric cancer (EBVaGC) is a subset of gastric adenocarcinoma that exhibits distinct clinicopathologic characteristics. It locates preferentially in the upper third of the stomach, with male sex predominance [1]. Histologically, it appears as “gastric carcinoma with lymphoid stroma,” which is characterized by irregular nests, cords, and sheets of poorly differentiated glands admixed with densely infiltrated lymphocytes [2]. Compared to other molecular subtypes suggested by The Cancer Genome Atlas (TCGA) group, EBVaGCs demonstrate a favorable prognosis [3]. EBVaGCs have been consistently reported to exhibit a good response to immune checkpoint inhibitors, as suggested by their high tumor-infiltrating lymphocytes [4]. Molecular analyses revealed that EBVaGCs underwent frequent *PIK3CA* and *ARID1A* mutations, *PD-L1* amplification, and a rare *TP53* mutation [5].

Recently, we encountered an unusual case of gastric cancer with distinct EBV-positive and -negative tumor areas, which has been usually described in the literature as a collision tumor [6–9]. Furthermore, we performed whole exome sequencing (WES) on both EBV-positive and -negative tumor areas and investigated their molecular characteristics. To the best of our knowledge, this is the first case wherein WES was performed for gastric cancer showing EBV-positive and -negative collision tumor-like histology.

## Case presentation

A 70-year-old man was referred to our hospital in November 2018 due to an impression of stomach cancer detected during a routine health check-up. He had no significant past medical history. On performing esophagogastroduodenoscopy, a 2.7-cm-sized Borrmann type 3 mass was found to be located at the greater curvature of the proximal body. Subsequently, a biopsy of the above-mentioned mass was performed, and the diagnosis of tubular adenocarcinoma was made. Abdominal computed tomography (CT) and positron emission tomography–CT revealed no signs of regional lymph nodes or distant metastasis. The patient agreed to undergo gastric surgery and we performed a distal gastrectomy and lymph node dissection to treat gastric cancer.

**Fig. 1:**
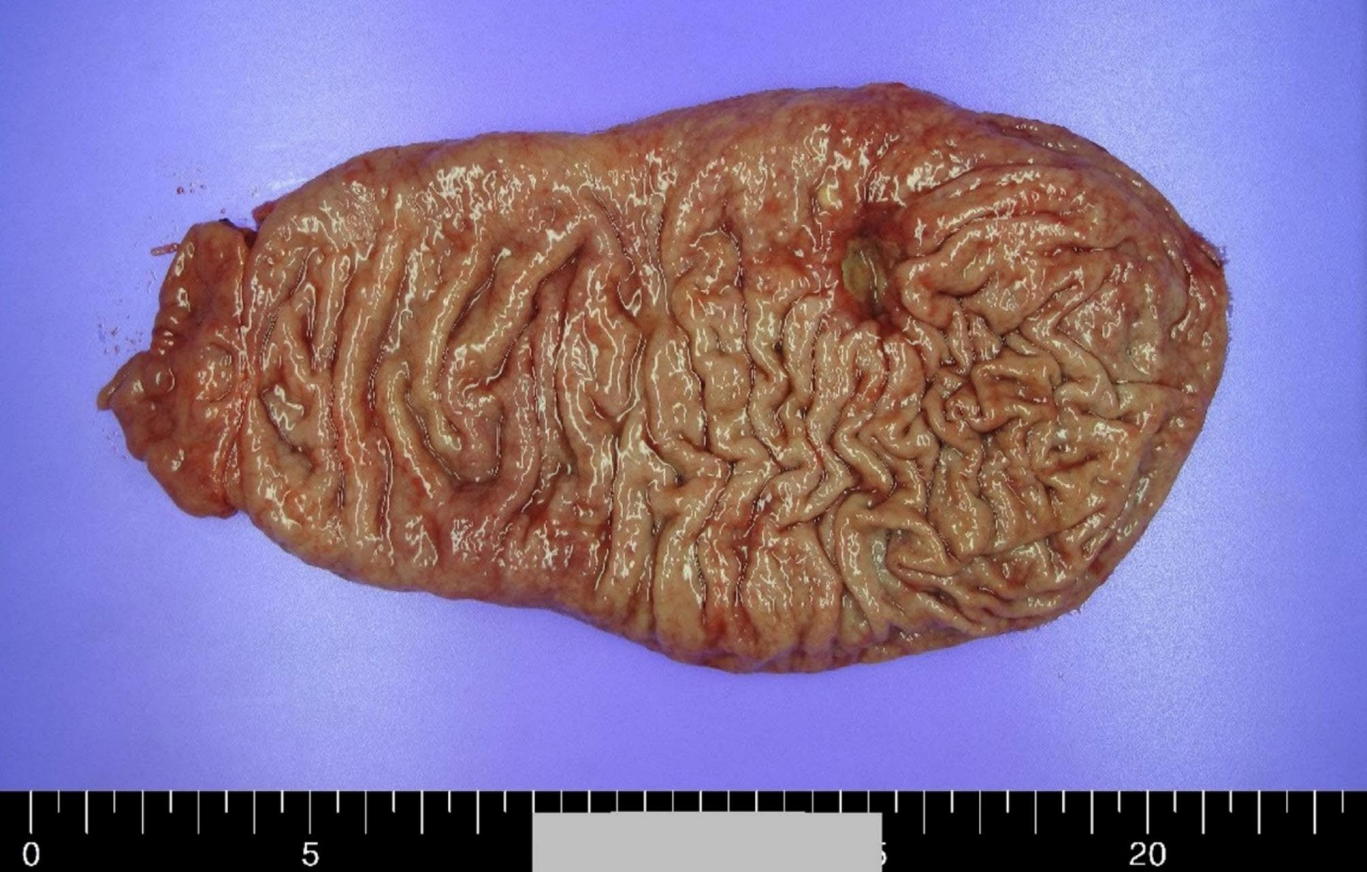
Gross appearance of the gastric cancer specimen. This distal gastrectomy specimen was opened along with the lesser curvature. Borrmann type 3 tumor was located in the greater curvature of the proximal body

**Fig. 2:**
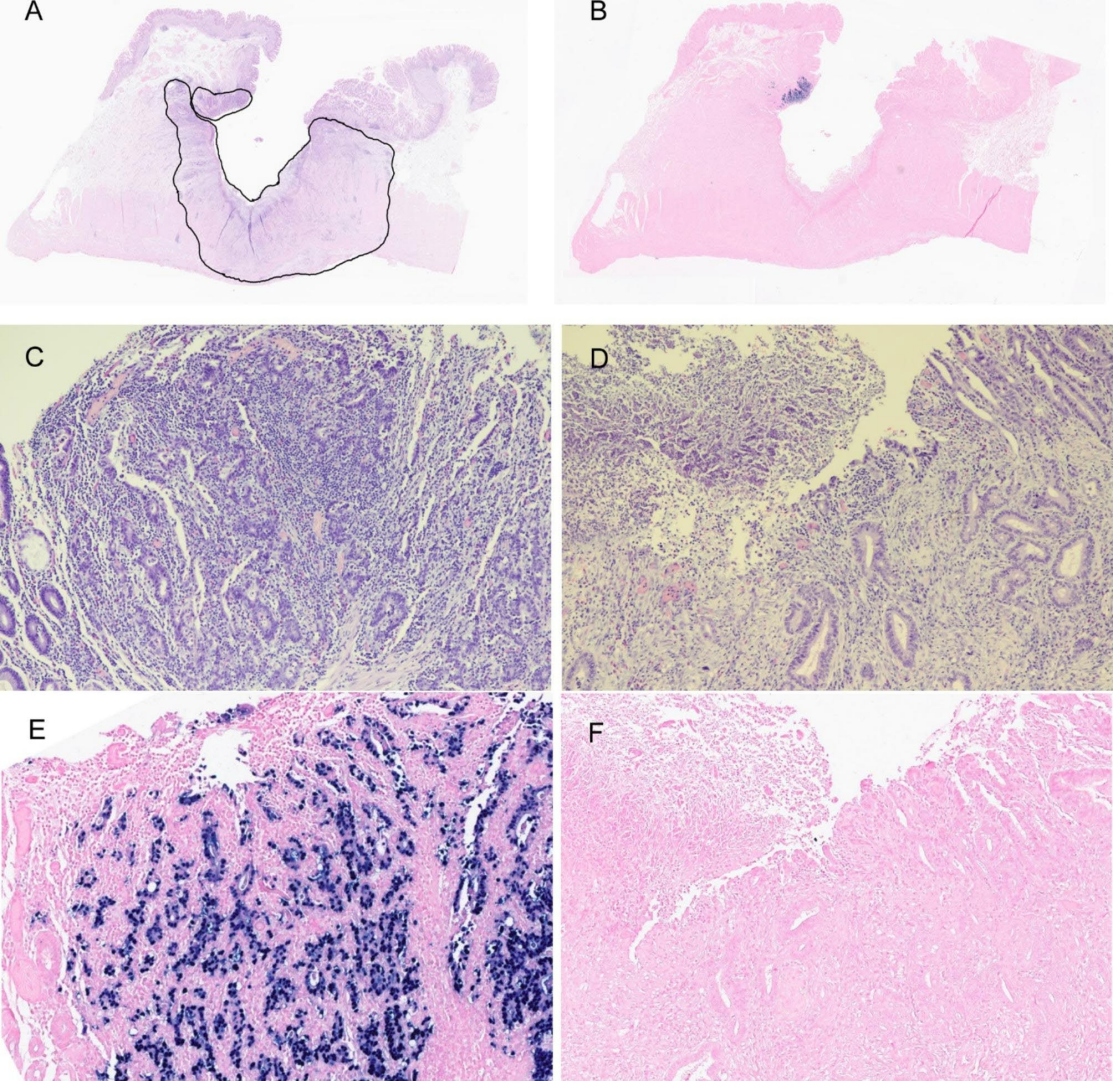
Representative images of gastric cancer with distinct EBV-positive and -negative tumor area. Loupe view of hematoxylin and eosin staining image (**A**) and EBER in situ hybridization result (**B**). At high magnification, (**C**) the EBV-positive tumor area shows less differentiated histology with increased lymphocytic infiltration, and (**D**) the EBV-negative tumor area shows conventional tubular adenocarcinoma histology with lesser degree of lymphocytic infiltration. (**E**) and (**F**) are EBER in situ hybridization results that correspond to (**C**) and (**D**), respectively. Original magnifications: (**C**)–(**F**): ×100

**Fig. 3:**
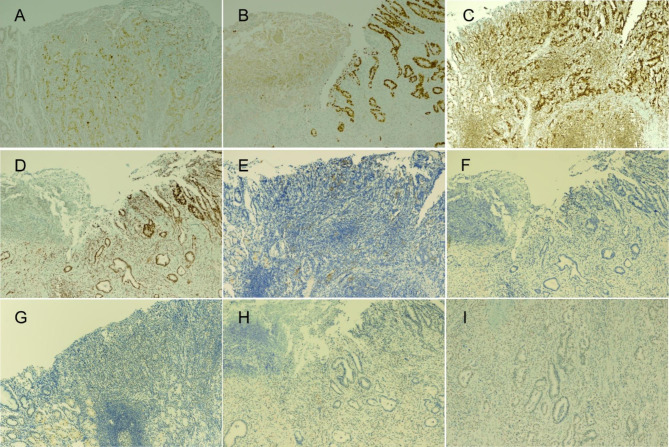
Immunostaining results of EBV-positive and -negative tumor areas. Immunostaining results for p53 ((**A**) and (**B**)), MLH1 ((**C**) and (**D**)), PD-L1 ((**E**) and (**F**)), and ARID1A ((**G**)–(**I**)) are shown. (**H**) is EBV-negative tumor area with loss of ARID1A expression, whereas (**I**) is EBV-negative tumor area with no loss of ARID1A expression. (**A**), (**C**), (**E**), and (**G**) correspond to EBV-positive tumor area shown in Fig. 2C, and (**B**), (**D**), (**F**), and (**H**) correspond to EBV-negative tumor area shown in Fig. 2D

Gross examination of the specimen revealed an ulcerative mass with an infiltrative border in the proximal body of the stomach (Fig. 1). On the cut section, the mass appeared to involve the subserosal layer. Histologic examination showed that the tumor consisted of moderately differentiated tubular adenocarcinoma with mild-to-moderate amount of lymphocytic infiltration infiltrating the subserosal layer; however, a small portion of the tumor located at the margin displayed less differentiated histology, and it was covered with dense lymphoid stroma (Fig. 2). Lymphovascular invasion or perineural invasion was not identified. Neither lymph node metastasis nor distant metastasis was identified (pT3N0M0). EBV-encoded RNA (EBER) in situ hybridization was performed following the EBER Probe Assay Protocol (Ventana Medical Systems Inc., Oro Valley, AZ, USA). Remarkably, EBER in situ hybridization result was positive for the focal tumor area with less differentiated histology and dense lymphoid stroma (approximately 10%) on one side, but the result was negative for the remaining tumor area with tubular adenocarcinoma histology (Fig. 2). In line with previous studies [5, 10], p53 immunohistochemistry (mouse monoclonal, clone DO7, 1:300; Novocastra, Newcastle, UK) revealed that the EBV-positive and -negative tumor areas exhibited wild-type and mutant-type p53 immunostaining patterns, respectively (Fig. 3A and B). P53 immunostaining pattern was interpreted either as “mutant” if ≥ 60% tumor cells showed strong nuclear expression or there was complete absence of staining or as “wild-type” if the tumor cells had p53 expression levels between these two levels (1–59% staining) [11, 12].

Based on the results of histologic examination, p53 immunostaining, and EBER in situ hybridization, we classified the tumor as EBV-positive and -negative collision gastric cancer. To explore the genomic characteristics of the tumor, we performed WES on EBV-positive and -negative tumor areas with matched normal tissue (lymph node tissue). Somatic mutation calling, tumor mutational burden, copy number variation, and mutational signature were analyzed based on the method described in previous studies [13, 14]. Briefly, sequencing was performed at an average depth of 235×. We identified 281 somatic single nucleotide variants (SNVs) and 375 small insertion and deletion (Indel) mutations in the EBV-positive and -negative tumor areas. The TMB were 3.27 muts/Mb and 3.35 muts/Mb for the EBV-positive and -negative tumor areas, respectively.

**Fig. 4:**
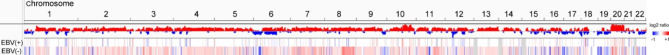
Copy number analysis of EBV-positive and EBV-negative tumor areas. Log2 ratios of tumor reads to normal reads are shown

Table 1 shows the pathogenic mutations found in EBV-positive and -negative areas. In particular, *ARID1A* p.S1488fs*, *KCNJ2* p.T75M, and *RRAS2* p.G23V were detected in both EBV-positive and -negative areas. *TP53* mutation was found only in the EBV-negative area, consistent with p53 immunostaining results. Remarkably, 32.7% of EBV-positive and 24.5% of EBV-negative areas shared 92 somatic SNVs and small Indel mutations, respectively. Copy number analysis revealed that the EBV-positive area showed frequent copy number alterations along the whole chromosome level compared to EBV-negative area (Fig. 4). There was no amplification of *CD274* (*PD-L1*) or *PDCD1LG2* (*PD-L2*) gene on both components. Mutational signature analysis demonstrated that the EBV-positive area had mutational signatures, single base substitution (SBS) 1, SBS4, SBS5, SBS17b, SBS19, SBS21, SBS29, SBS32, SBS42, and SBS57, and the EBV-negative area had SBS1, SBS2, SBS5, SBS9, SBS19, SBS20, SBS37, SBS38, SBS41, SBS45, SBS49, SBS53, SBS55, SBS58, and SBS59.

**Table 1:** Pathogenic genomic variants found in EBV collision gastric cancer

Tumor area.	Chromosome	Position	Gene	Variant type	HGVS.c	Protein change	VAF
EBV-positive	Chr1	26,774,684	*ARID1A*	Frameshift mutation	c.4458_4477delGGCATCAGCTGAGGTTGCTC	p.Ser1488fs	0.059
EBV-positive	Chr17	70,175,263	*KCNJ2*	Missense mutation	c.224 C > T	p.Thr75Met	0.083
EBV-positive	chr11	14,358,803	*RRAS2*	Missense mutation	c.68G > T	p.Gly23Val	0.067
EBV-positive	chr15	44,715,574	*B2M*	Frameshift mutation	c.220_233delTTGTCTTTCAGCAA	p.Leu74fs	0.012
EBV-positive	Chr6	29,944,149	*HLA-A*	Frameshift mutation	c.650_651dupCC	p.Ile218fs	0.027
EBV-positive	chrX	45,089,874	*KDM6A*	Nonsense mutation	c.3836G > A	p.Trp1279*	0.139
EBV-negative	Chr1	26,774,684	*ARID1A*	Frameshift mutation	c.4458_4477delGGCATCAGCTGAGGTTGCTC	p.Ser1488fs	0.126
EBV-negative	Chr17	70,175,263	*KCNJ2*	Missense mutation	c.224 C > T	p.Thr75Met	0.203
EBV-negative	chr11	14,358,803	*RRAS2*	Missense mutation	c.68G > T	p.Gly23Val	0.175
EBV-negative	Chr3	179,218,294	*PIK3CA*	Missense mutation	c.1624G > A	p.Glu542Lys	0.078
EBV-negative	chr17	7,673,803	*TP53*	Missense mutation	c.817 C > T	p.Arg273Cys	0.272

We further analyzed the immunohistochemistry of MLH1 (mouse monoclonal, prediluted, clone M1, Roche, Basel, Switzerland), PD-L1 (mouse monoclonal, clone 22C3, 1:50; Agilent, CA, USA), and ARID1A (rabbit polyclonal, 1:400; Sigma-Aldrich, MO, USA), on both components. MLH1 immunohistochemistry revealed no loss of nuclear expression for both components (Fig. 3C and D). EBV-positive area had higher PD-L1 combined positive score (CPS) than EBV-negative area (CPS 10 vs. CPS 1). PD-L1 tumor proportion score was negative for both components (Fig. 3E F). EBV-positive area showed no loss of ARID1A expression (Fig. 3G), whereas EBV-negative area showed loss of ARID1A expression on 60% of tumor cells (Fig. 3H and I).

After the surgery, the patient received eight cycles of tegafur/gimeracil/oteracil combination oral chemotherapy (TS-1) for 10 months. During follow-up, abdomen CT was performed at a one-year interval, and its findings did not reveal any sign of metastasis or recurrence. The patient was alive without any symptoms for 52 months postsurgery.

## Discussion and conclusions

We presented the case of gastric cancer with distinct EBV-positive and -negative tumor areas and further investigated their genetic characteristics. Since the tumor had EBV- positive and -negative areas at each side with distinct histology and p53 immunostaining pattern, we first considered this case as EBV collision gastric cancer. EBV collision gastric cancers have been rarely reported in the scientific literature [6–9]. Intratumoral heterogeneity of EBVaGCs have been reported in a few previous studies [15, 16]. However, EBV collision gastric cancer, unlike gastric cancer with heterogeneous EBV positivity, has distinct tumor components at the border that exhibit EBV positivity and negativity, rather than having two intermixed components. Miyabe et al. suggested that EBV-positive and -negative collision gastric cancers have different histogenesis based on the result of *TP53* sequencing, targeted next-generation sequencing, and *HER2* and *C-MYC* fluorescence in situ hybridization (FISH) study [6].

Interestingly, the WES results in this case report suggested that morphologically EBV collision gastric cancer with distinct p53 immunostaining pattern can be clonally related, as distinct EBV-positive and -negative areas in this case share a significant number of somatic genetic alterations, including pathogenic mutations of *ARID1A*, *KCNJ2*, and *RRAS2*. Although EBV-positive and -negative gastric cancers share a significant portion of genetic mutations that may have occurred in early tumorigenesis, they also had distinct pathogenic alterations, which appear to be a subclonal event. The EBV-positive tumor area lacks the *TP53* mutation, whereas the EBV-negative area exhibits the *TP53* mutation. EBVaGCs rarely exhibit *TP53* mutation, but it occurs frequently in the conventional intestinal-type adenocarcinoma [17]. In addition, pathogenic alterations of *HLA-A*, *B2M*, and *KDM6A* were identified in the EBV-positive area, and alteration of *PIK3CA* was identified in the EBV-negative area. Copy number analysis revealed that the EBV-negative area had complex copy number alterations compared to EBV-positive area. This is also consistent with previous studies showing that gastric cancer with intestinal histology (molecularly chromosomally instable type according to TCGA classification) frequently has copy number aberrations [5, 18]. Mutational signature analysis also revealed that both EBV-positive and -negative areas had common signatures of SBS1, SBS5, and SBS19, although different signatures were detected in each area.

In a recent study, Kondo et al. [19] suggested that EBV loss may occur during tumor progression, which can affect the immune evasion mechanism. Therefore, it is possible that EBV-negative component in this case would have developed due to “EBV drop-off” during tumor progression. Another possible explanation could be that initial progenitor tumor clones without EBV infection (which shared some pathogenic mutations including *ARID1A*) subsequently acquired EBV infection during the tumor progression. As we did not perform additional tests such as ultrasensitive digital droplet PCR or *TP73* methylation analysis that can detect traces of EBV or previous EBV infection [19, 20], we could not determine which of these two scenarios was relevant in this case. However, the pathogenic mutation of *ARID1A*, which typically develops in EBVaGC or MSI-high gastric cancer [5], favors the possibility of the former. Although immunohistochemistry showed loss of ARID1A expression only in the partial area of EBV-negative component, discrepancy between ARID1A immunostaining and sequencing have been reported in previous literature [21, 22]. Considering the read depth and variant allele frequency (322×, 5.9% and 175×, 12.6% for EBV-positive and -negative tumor areas), it is unlikely that *ARID1A* p.S1488fs* in this case is false positive mutation.

The results of genetic analysis suggested that morphologically gastric EBV collision tumor with distinct EBV-positive and -negative areas can be clonally related, although previous gastric EBV collision cancer cases supported the notion that they are of different origin. Further research is warranted to determine whether this is an exceptionally rare case or distinct EBV-positive and -negative gastric morphologically collision cancers can share genetic alterations more frequently than previously thought.

## Data Availability

The datasets used and/or analyzed during the current study are available from the corresponding author on reasonable request.

## List of Abbreviations

CT: Computed tomography
EBER: Epstein–Barr virus-encoded RNA
EBV: Epstein–Barr virus
EBVaGC: Epstein–Barr virus-associated gastric cancer
Indel: Insertion and deletion
SBS: Single base substitution
SNV: Single nucleotide variant
TCGA: The Cancer Genome Atlas
WES: Whole exome sequencing

## References

[1] Yang J (2020). Epstein-Barr virus-associated gastric cancer: a distinct subtype. Cancer Lett.

[2] Cheng N (2015). Is gastric lymphoepithelioma-like carcinoma a special subtype of EBV-associated gastric carcinoma? New insight based on clinicopathological features and EBV genome polymorphisms. Gastric Cancer.

[3] Sun K (2020). EBV-Positive gastric Cancer: current knowledge and future perspectives. Front Oncol.

[4] Zavros Y, Merchant JL (2022). The immune microenvironment in gastric adenocarcinoma. Nat Rev Gastroenterol Hepatol.

[5] Cancer Genome Atlas Research (2014). Comprehensive molecular characterization of gastric adenocarcinoma. Nature.

[6] Miyabe K (2021). Collision of Epstein-Barr virus-positive and -negative gastric cancer, diagnosed by molecular analysis: a case report. BMC Gastroenterol.

[7] Matsuda I (2015). A case of gastric cancer with heterogeneous components of EB virus (+)/TP53 (+) and EB virus (-)/TP53 (-). Int J Clin Exp Pathol.

[8] Aoyama H (2015). Collision tumor involving gastric carcinoma with lymphoid stroma and moderately differentiated adenocarcinoma. J Japan Surg Assoc.

[9] Okada A (2010). Gastric collision tumor of adenocarcinoma and Epstein–Barr virus–related carcinoma–a case report–. J Japan Surg Assoc.

[10] Chang MS (2001). Clinicopathologic characteristics of Epstein-Barr virus-incorporated gastric cancers in Korea. Pathol Res Pract.

[11] Lee SH (2013). Genetic alteration and immunohistochemical staining patterns of ovarian high-grade serous adenocarcinoma with special emphasis on p53 immnnostaining pattern. Pathol Int.

[12] Yemelyanova A (2011). Immunohistochemical staining patterns of p53 can serve as a surrogate marker for TP53 mutations in ovarian carcinoma: an immunohistochemical and nucleotide sequencing analysis. Mod Pathol.

[13] Kim M (2022). Genomic characteristics of invasive mucinous adenocarcinoma of the lung with multiple pulmonary sites of involvement. Mod Pathol.

[14] Alexandrov LB (2020). The repertoire of mutational signatures in human cancer. Nature.

[15] Kim HN, Ahn S, Kim KM (2022). Gastric cancer with Epstein-Barr virus heterogeneity: evaluation of the frequency, clinicopathologic features, and genomic profiles. Pathol Res Pract.

[16] Böger C (2017). Epstein-Barr virus-associated gastric cancer reveals intratumoral heterogeneity of PIK3CA mutations. Ann Oncol.

[17] Kim M, Seo AN (2022). Molecular Pathology of Gastric Cancer. J Gastric Cancer.

[18] Nakamura Y (2021). Biomarker-targeted therapies for advanced-stage gastric and gastro-oesophageal junction cancers: an emerging paradigm. Nat Rev Clin Oncol.

[19] Kondo A (2023). Loss of viral genome with altered immune microenvironment during tumour progression of Epstein-Barr virus-associated gastric carcinoma. J Pathol.

[20] Siciliano MC (2022). EBV persistence in gastric cancer cases conventionally classified as EBER-ISH negative. Infect Agent Cancer.

[21] Wiegand KC (2010). ARID1A mutations in endometriosis-associated ovarian carcinomas. N Engl J Med.

[22] Khalique S (2018). Optimised ARID1A immunohistochemistry is an accurate predictor of ARID1A mutational status in gynaecological cancers. J Pathol Clin Res.

